# Hybrid Pipeline Hardware Architecture Based on Error Detection and Correction for AES

**DOI:** 10.3390/s21165655

**Published:** 2021-08-22

**Authors:** Ignacio Algredo-Badillo, Kelsey A. Ramírez-Gutiérrez, Luis Alberto Morales-Rosales, Daniel Pacheco Bautista, Claudia Feregrino-Uribe

**Affiliations:** 1 CONACYT-Instituto Nacional de Astrofísica, Óptica y Electrónica, Puebla 72840, Mexico; algredobadillo@inaoep.mx (I.A.-B.); kramirez@inaoe.mx (K.A.R.-G.); 2 Faculty of Civil Engineering, CONACYT-Universidad Michoacana de San Nicolás de Hidalgo, Morelia 58000, Mexico; 3 Departamento de Ingeniería en Computación, Universidad del Istmo, Campus Tehuantepec, Oaxaca 70760, Mexico; dpachecob@bianni.unistmo.edu.mx; 4 Instituto Nacional de Astrofísica, Óptica y Electrónica, Puebla 72840, Mexico; cferegrino@inaoep.mx

**Keywords:** error detection, error correction, decision tree, pipeline architecture, AES security

## Abstract

Currently, cryptographic algorithms are widely applied to communications systems to guarantee data security. For instance, in an emerging automotive environment where connectivity is a core part of autonomous and connected cars, it is essential to guarantee secure communications both inside and outside the vehicle. The AES algorithm has been widely applied to protect communications in onboard networks and outside the vehicle. Hardware implementations use techniques such as iterative, parallel, unrolled, and pipeline architectures. Nevertheless, the use of AES does not guarantee secure communication, because previous works have proved that implementations of secret key cryptosystems, such as AES, in hardware are sensitive to differential fault analysis. Moreover, it has been demonstrated that even a single fault during encryption or decryption could cause a large number of errors in encrypted or decrypted data. Although techniques such as iterative and parallel architectures have been explored for fault detection to protect AES encryption and decryption, it is necessary to explore other techniques such as pipelining. Furthermore, balancing a high throughput, reducing low power consumption, and using fewer hardware resources in the pipeline design are great challenges, and they are more difficult when considering fault detection and correction. In this research, we propose a novel hybrid pipeline hardware architecture focusing on error and fault detection for the AES cryptographic algorithm. The architecture is hybrid because it combines hardware and time redundancy through a pipeline structure, analyzing and balancing the critical path and distributing the processing elements within each stage. The main contribution is to present a pipeline structure for ciphering five times on the same data blocks, implementing a voting module to verify when an error occurs or when output has correct cipher data, optimizing the process, and using a decision tree to reduce the complexity of all combinations required for evaluating. The architecture is analyzed and implemented on several FPGA technologies, and it reports a throughput of 0.479 Gbps and an efficiency of 0.336 Mbps/LUT when a Virtex-7 is used.

## 1. Introduction

In an emerging automotive environment where connectivity is a core part of autonomous and connected cars, it is essential to guarantee secure communications both inside and outside the vehicle. Today cars are equipped with several gadgets to make the driving experience more comfortable, such as links to cellular data, infotainment systems, mobile apps, GPS, etc., where data can be compromised by many factors including noise in the transmission channel, systems failure, temperature changes, or outside attacks. Modern communications systems are now, more than ever, the target of attackers and susceptible to errors during transmission. Encryption algorithms have been widely applied to communications systems to guarantee data security, as is the case of the Advanced Encryption Standard (AES), which is a symmetric block cipher based on substitution-permutation chosen by the US government to be a federal standard. The AES algorithm has been applied in several applications, such as satellite communications, file encryption, processor security, and many others.

Recently, AES has also found a place in automotive developments, for reasons such as guaranteeing privacy in communication [1], real-time authentication on automotive systems [2], and preventing keyless entry on automobile remote keyless systems [3,4]. Particularly, AES algorithms have been widely applied to protect communications in onboard networks and outside the vehicle. Lugo-Meneses et al. in [5] created the Secure AES Frame Encryption (SAFE) kernel as part of the Electronic Control Unit (ECU) and implemented the AES algorithm to cipher CAN FD messages to avoid hacking the ECU. The EVITA project co-founded by the European Union aims to improve the security of the automotive onboard network to attacks from new V2X applications as well as those performed by having physical access to the vehicle [6], in which its cryptographic building block AES-128 is incorporated. In addition, Jattala et al. in [7], secure the communication channel SMS of SATS (secure automotive telematics systems) using an AES-256 encryption algorithm. A hardware implementation of the Advanced Encryption Standard (AES) algorithm with a 32% higher speed throughput than the state of art is presented by [8].

Basically, AES offers only confidentiality, although it can be employed to offer other security services such as authentication and integrity. The algorithm itself does not guarantee the correct internal calculations and failure avoidance. For example, in hardware architectures where there is noise and interference, the algorithm cannot overcome faults. Some studies about how noise causes failures on a field-programmable gate array (FPGA) have been developed. For example, Traoré et al. [9] identify each noise source in a giant magneto-impedance (GMI) sensor based on an FPGA, predicting the equivalent magnetic noise level of the sensor in the white noise region. An investigation conducted by Toan et al. [10] demonstrated that variance in the voltage supply can decrease the immunity of the I/O buffer by about 16.8 dB and also showed that under the conducted noise in the frequency domain, an FPGA chip with a redundancy-based fault-tolerant circuit can improve its immunity. Benfica et al. [11] described a methodology to analyze the single-event upset (SEU) sensitivity of FPGA devices to combined effects of conducted electromagnetic interference (EMI) and total-ionizing radiation (TID), demonstrating that noise on power bus pins is more harmful to SEU cross-section than $V_{DD}$ reductions. Shum et al. [12] determined that an algorithm for glitch power reduction in a commercial FPGA decreased 13.7%. Kotipalli et al. in [13] present the design, hardware implementation, and performance analysis of an asynchronous AES key expander and round function resistant to a side-channel attack (SCA); its benefits include reduced and uniform switching activities and a reduced signal-to-noise (SNR) ratio. An approach that applies SHA as a software countermeasure to mitigate SCA attacks was developed by Frieslaar et al. [14], and the results showed that the entire AES-128 key was recovered from a CPA attack.

Previous studies presented by Biham and Shamir [15] proved that implementations of secret key cryptosystems such as AES in hardware are sensitive to differential fault analysis. In addition, Bertoni et al. in [16] demonstrated that even a single fault during encryption or decryption could cause a large number of errors in encrypted or decrypted data. Therefore, it is necessary to develop fault detection techniques to protect AES encryption and decryption processes. When transmitting in an insecure and noisy channel, encryption and error correction cannot be separated [17]. During encryption and decryption, it is crucial to guarantee that data have not been corrupted. Hence, the main task discussed in this paper is detecting and correcting system-level errors and faults.

We can implement cryptographic solutions in software or hardware. Nevertheless, there is a trend in research for embedded systems applying IoT and IIoT to secure communications for automotive systems [1,2,3,4,5,6,7,8]. Hardware implementations use techniques such as iterative, parallel, unrolled, and pipeline architectures. Moreover, it is essential to highlight that each has different advantages and disadvantages (see [18] for a deeper analysis). Hence, this motivates us to examine pipelined architectures to obtain gains from using the same hardware resources to process several data blocks at different times focused on redundancy in the cryptographic calculation, in this way detecting and correcting errors if possible.

In hardware design, a pipeline structure is a set of data processing elements connected in stages and series so that the output of one stage is the input of the next one. Executing these elements in parallel or in a time-sliced fashion requires some buffer storage (pipeline registers) inserted in between stages. A pipeline structure typically requires more resources (circuit elements, processing units, computer memory, etc.) than one that executes one batch at a time because each pipeline stage cannot reuse the resources of the other stages. We can obtain highly concurrent implementations by overlapping the execution of consecutive iterations. Forward and backward scheduling is iteratively used to minimize the delay to have more silicon area for allocating additional resources, which will increase throughput. Key pipeline parameters are the number of pipeline stages, latency, clock cycle time, delay, turnaround time, and throughput. Thus, we can constrain a pipeline synthesis problem either by resource or time or a combination of both [19]. In this case, the objective is to maximize the performance, given available resources. Additionally, a time-constraint pipeline synthesis specifies the required throughput and turnaround time, with the objective of the scheduler being to find a schedule that consumes the minimum of resources. Finally, retiming is applied to exploit the ability to move registers in the circuit to decrease the length of the longest path while preserving its functional behavior. Therefore, the analysis should focus on improving the performance by maximizing the clock frequency and constraining the number of registers for a target clock period

The design and development of pipeline architectures are not direct. These processes need analysis and design evaluations, motivating the present research to solve the detection and correction of errors in the implementations of the algorithm AES inside a pipeline hardware architecture, considering an inference system that allows determining if the exit is correct. Furthermore, the consideration of balancing a high throughput, low power consumption, and fewer hardware resources in the pipeline design is challenging.

Therefore, this paper proposes a novel hardware architecture based on a hybrid hardware-efficient approach for an AES cryptographic algorithm focusing on error and fault detection. The main contributions of this paper are as follows:

- The hybrid architecture combines both the concept of hardware and time redundancy through a pipeline structure, analyzing and balancing the critical path, and distributing the processing elements within each stage.
- From the perspective of the pipeline design, all risks and conflicts in the data transmission, through registers within each stage, are eliminated due to the processing of an iterative algorithm such as cryptographic ones that require giving too many passes to the data to generate its final output.
- A pipeline structure for ciphering five times on the same data blocks with a voting module to verify when an error occurs or to output correct cipher data.
- The voting module is optimized using a decision tree to reduce the complexity of all combinations required for evaluating when correct cipher data are present in the five registers of the pipeline structure.
- An analysis for the design, implementation, and selection of the architecture is implemented on several FPGA technologies such as Spartan-7, Artix-7, Kintex-7, and Virtex-7, where the best results are reached when the architecture is implemented on Virtex-7, reporting a throughput of 0.479 Gbps and efficiency of 0.336 Mbps/LUT.

The paper is organized as follows: Section 2 presents the state of art on error detection and correction, Section 3 presents some important concepts applied in the proposed hardware architecture, and Section 4 explains the reinforced architecture that balances high performance and low power consumption and fewer hardware resources. In Section 5, the results of the implementation and the comparisons are shown. Finally, we present conclusions in Section 6.

## 2. Background

### 2.1. Advanced Encryption Standard

Cryptographic algorithms used for confidentiality are divided into stream ciphers and block ciphers. The Advanced Encryption Standard (AES) is considered a block cipher based on substitution-permutation, which became a standard of the US National Institute of Standards and Technology (NIST) in 2001. Through modes of operations, this algorithm can be used to provide other security services or be used as a stream cipher.

The AES algorithm consists of three procedures: encryption, decryption, and key expansion. In the encryption process, first, a key mixing phase is performed where the initial key is added to the initial entry. Then ten rounds of four transformations are done [20]: *SubBytes:* a non-linear substitution of bytes; *ShiftRows:* a byte rotation of the S state rows using an offset that depends on the row itself; *MixColumns:* a linear algebraic transformation of each column of the state, over the Galois field; and *AddRoundKey:* a bit-wise XOR operation of the current round key, provided by the key schedule to state S. Each round key can also be seen as a square matrix. The key schedule updates the array by using some of the encryption operations: in particular, each byte of the last row of the array is fed as an input to the SubBytes operation [21]. A pseudo-code for the AES encryption algorithm is presented in Algorithms 1 and 2.
**Algorithm 1:** AES Encryption Algorithm [22]$Block_{in}$, round_keyState**for***i* = 1 $N_{r}-1$ **do** SubBytes(state) ShiftRows(state) MixColumns(state) AddRoundKey(state,round_key[i])**end for**SubBytes(state)ShiftRows(state)$AddRoundKey(state,round\_key\left[N_{r}\right])$

**Algorithm 2:** Decision Tree
TreeGeneration (Sample,Feature)

**if** StopCondition(Sample,Feature)=True **then**


 Leaf = NodeCreation()


 LeafLabel = Classify(Sample)

  **return**  Leaf

**end if**


Root = NodeCreation()


RootConditionTest = FindSplit(Sample,Feature)

V = {v∣v a possible output of RootConditionTest}

**for** each v **do**

$\;S_{v}\;=\;\{s|RootConditionTest\left(Sample\right)=v$ and s ϵ S}
  child = $TreeGeneration(S_{v},Feature)$
 Add child as a descent of root

**end for**

**return** Root


### 2.2. FPGA Optimization Techniques

Optimization techniques play a significant role in achieving suitable hardware implementations, mainly focused on balancing a high throughput, low power consumption, and fewer hardware resources in the FPGA design. In this sense, Kalaiarasi et al. in [23] divide FPGA optimization techniques by the application requirements to be optimized: speed, area, and power.

- Speed Optimization: This optimization may have three approaches: (1)*Pipelining*, which improves the maximum clock frequency and is performed by adding registers to the critical path; (2)*Parallel Processing*, where, multiple parallel outputs are calculated over a clock period; and (3)*Register Balancing* applied to fulfill design-time requirements by redistributing logic evenly between registers.
- Area Optimization: This focuses mainly on two strategies: *Resource Sharing*, which employs a sole functional block to run many operators, and *Proper Reset Strategy*, in which the first effect on the area has to do with the set/reset condition for every flip-flop, but bringing a reset into every synchronous element can cause the synthesis and mapping tools to push the logic into a coarser implementation [24].
- Power Optimization: There are two ways in which FPGAs dissipate power, dynamically and statically. The first occurs during the charging and discharging of internal capacitances, and the second one by leaking currents during device standby [25].

## 3. Related Work

### 3.1. Error Detection and Correction Status

An error occurs when the output information of some process does not match the expected information, which can be caused during data transmission by third-party sources. Moreover, noise can introduce errors in the binary sequences that travel from one system to another. Moreover, while encrypting a message, it can be changed during the process; therefore, wrong authentication codes are added to the ciphertext. Hence, this would cause a bit 0 to change to 1 or a bit 1 to change to 0. Generally, some approaches to solve error detection in hardware design are adopted, such as:

- Time redundancy, which seeks to reduce the amount of hardware while sacrificing additional time. The main drawback with time redundancy is the assumption that the data necessary to repeat a calculation are available in the system [26].
- Information redundancy can overcome data errors that may occur when they are transferred or stored in a memory unit.
- Hardware redundancy/Physical Redundancy is obtained by including additional hardware in the design to identify or remove the effects of a failed component [27].

During data transmission, many errors can be generated, from physical defects to environmental interference. Panem et al. in [28] identify four error types in a communication channel that can alter data:

1. Noise or electrical distortion: sound waves, electrical signals, noise from motors, power switches, etc.
2. Burst errors: large sets of bits that happen when many interconnected bit errors are occurring at numerous points.
3. Random bit errors: occur when bits have been rearranged by accident in the transmission process.
4. Cross talk and echo: when the transmission cable is environed by other transmission lines.

The error detection mechanism aims to protect applications against various types of errors and faults. The main goal is to ensure that every single bit of error can be detected and corrected.

Several authors have proposed different approaches to solve error detection and correction status. Anton et al. in [29] implemented an error detection and correction circuit using associative memories, providing a high-speed response. They accomplish the correction by matching the received word code with one of the stored ones, choosing it in Hamming distance.

Many authors have proposed hardware implementations of AES error detection and correction. Kamboj et al. in [30] proposed an algorithm to detect a soft error in AES cipher output during the hardware implementations. On the other hand, an execution error detection and correction algorithm are carried out by mapping ShiftRow and MixColumn. This solution uses the cipher’s linear operations, involving XOR and shift to detect errors in the merged transformations [31]. A data acquisition system is proposed by Mandal et al. in [32] which supports high-speed data communication and reaches multi-bit error correction. An error correction procedure for the Advanced Encryption Standard (AES) is proposed by Biernat et al. in [33]: a prediction for each AES transformation is implemented, detecting all bit errors of odd order and most bit errors of even order injected into a single bit of the data block.

A balance among high throughput, low power consumption, and fewer hardware resources is fulfilled by our proposed architecture, presented in the next section. The design and analysis are essential due to cryptography requirements in many real applications (focusing on Industry 4.0, IoT, IIoT, and vehicles), where noise situations and interference can cause errors or faults.

### 3.2. Fault Tolerance and Other Concepts

Fault tolerance (FT) is defined by [34] as the ability of a system to operate normally given the presence of malfunctioning resources or faults. Shang et al. in [35] classify failure modes as:

- Transient: normally caused by transitory events (mechanical vibration, voltage fluctuation, etc.). The system can recover in a short time.
- Pseudo-permanent: usually caused by transient accidents that happened in SRAM cells. These faults are similar to permanent failures but can be recovered by reloading the system.
- Permanent: habitually generated by physical damages to the chip (manufacturing defects, electromigration, etc.). The system can be recovered by loading a proper alternative configuration.

The goal of fault tolerance is to maintain the correct transmission in the presence of (1) active faults, (2) limiting the impact of failures, and (3) transparently tolerating problems. The first item concerns the probability that the system operates correctly throughout a complete interval of time. The second is related to the probability that the system is operating correctly at the time *t* [36]. The last item is focused on increasing reliability and availability. Availability differs from reliability in that reliability involves an interval of time, while availability is taken at an instant of time [37].

For a system to be fault-tolerant, some resources need to be balanced (system performance, cost, and power), including error detection and recovery.

A lightweight hybrid fault-tolerant approach for AES, proposed by [38], integrates an algorithm based on a fault-tolerant (ABFT) technique and a fault-tolerant technique for the S-Box byte substitution operation; furthermore, the error detection procedure occurs at the end of all rounds applying for information redundancy. Kalaiarasi et al. in [23] present a model to detect and correct single event upsets in onboard implementations of the AES algorithm, based on the Hamming error-correcting code to satellite operation.

A fault-tolerant architecture called configurable fault-tolerant AES (CFTA), and its variants are presented in [39] for the AES block cipher with different key sizes and different pipeline levels to mitigate the reliability issue of secure architectures. It is a fault resilient architecture based on a modified temporal redundancy for the parallel implementation of AES. Kamal et al. in [40] implemented and compared the throughput and area overheads associated with parity-based error detection and (algorithm level, round level, and operation level) redundancy-based countermeasures. Finally, Sheikhpour et al. in [41] present two fault-tolerant hardware architectures, which modify the round path and divide it into two pipeline stages. The designs combine hardware and time redundancies, the first for the AES round function and the second for the AES key expansion unit’s hardware.

## 4. Proposed Hardware Architecture

The design of the hybrid-redundancy architecture has a pipeline structure based on five stages, where each stage is a set of data processing elements connected in series, which are executed in parallel and filled with the same data blocks in a time-sliced manner for independently computing five results (assuming that these will be equal). In applications where multiprocessors or multi-channels are required—for example, in works developed by Elkabbany et al. [42] and Nabil et al. [43]—a pipeline architecture increases the performance and throughput by processing independent communications lines; in our case, the pipeline architecture is used for processing the same data block, focusing on the detection and correction of errors. For this process, some buffer storage (pipeline registers) is inserted between the five data processing elements, IR&F (Initial Round and Feedback), S&S (SubBytes and ShiftRows), M (Mix Columns), A (Add Round Key), and IC (Intermediate Cipher Data). In this way, each pipeline stage cannot reuse resources of other stages. Therefore, the architecture implements more resources (memory, circuit elements, etc.) than a batch system. As mentioned, a comparison analysis was evaluated for selecting the five-stage architecture, evaluating the critical path of the design and balancing between the data to process and the latency. There are many metrics for evaluating the pipeline performance, and their selection depends on the applications, defining some problems and improving some parameters such as the number of pipeline stages, latency, clock cycle time, delay, turnaround time, efficiency, and throughput. In our case, the design of the architecture is based on an open problem in the pipeline synthesis, which is bounded either by resources, time, or energy consumption. A resource-constraint pipeline synthesis limits the area of a chip or the available number of functional units of each type, which was improved by adding the necessary registers for storing intermediate data among processing elements. A time-constraint pipeline synthesis specifies the required throughput and turnaround time to consume minimum resources. It is important to highlight that an essential concept in circuit pipelining is retiming [19], which exploits the ability to move registers in the circuit to decrease the length of the longest path while preserving its functional behavior [44,45]. The performance optimization problem of pipelined architectures is to maximize the clocking rate or equivalently minimize the cycle time of the circuit, which is reached by iteratively evaluating different processing elements between two-neighbor stages, where the aim of constrained min-area retiming is to constrain the number of registers for a target clock period. Under the assumption that all registers have the same area, the min-area retiming problem reduces to seeking a solution with the minimum number of registers in the circuit, all this being an iterative task, which provides the final proposed pipelined architecture.

**Figure 1:**
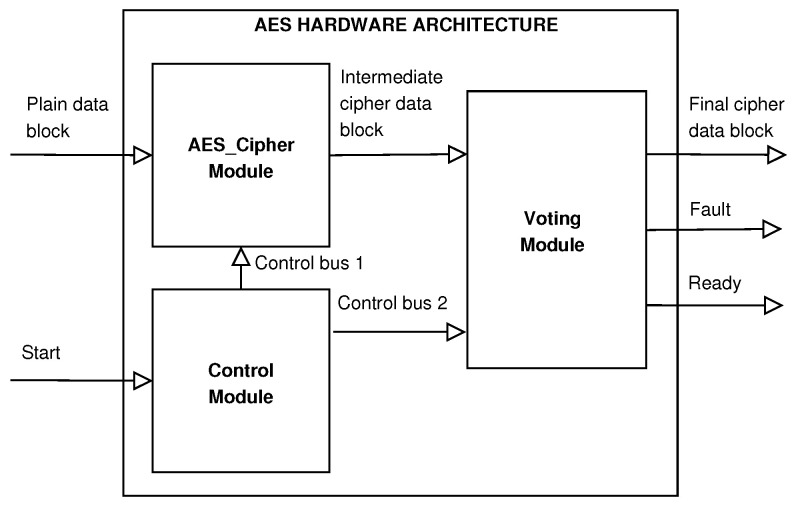
Block diagram of the proposed hardware architecture.

We implement the version of AES as a block cipher; therefore, in the hardware architecture, we divide the entire message into message blocks, and each block is stored and sent via the *Plain Data Block*. Each one is sent at a different time through the *Intermediate Encryption Data Block*, where they are compared and evaluated; if they are different, a failure occurs. The *Final Encryption Data Block* generates encrypted data at the output and a failure signal indicating whether the encrypted data are fault-free or not. We illustrate the block diagram of the proposed hardware architecture in Figure 1, which acquires and encrypts the data in several clock cycles.

Many processes execute complex operations such as cryptographic computations and decisions for selecting the correct cipher data if they exist. We divide the proposed hardware architecture into three modules: (a) *AES_Cipher*, which acquires and ciphers the data through a pipeline structure; (b) *Voting*, which stores, compares, and evaluates five stored intermediate blocks of cipher data; and (c) *Control*, which manages the data flow. The main idea for the design and development of the hardware architecture focuses on the characteristics of two approaches to fault detection revised in Section 3.2. In the first case, *Time Redundancy*, we use the same hardware for the cipher data several times, requiring more time to calculate and, consequently, consuming more power. However, fewer hardware resources are needed, and lower throughput is reached due to more significant latencies. In the second case, *Hardware Redundancy*, we provide high throughput at the cost of greater hardware resources and more power consumption than other proposals. The design can become complex, depending on the interconnection form of the different replicated modules for computing the same cipher data.

The proposed hardware implementation presents a novel architecture with a pipelined structure for providing time redundancy but using the same hardware by analyzing these approaches. Our proposal balances a high throughput, low power consumption, and fewer hardware resources. Next, we discuss the three modules of the hardware architecture.

### 4.1. AES_Cipher Module

**Figure 2:**
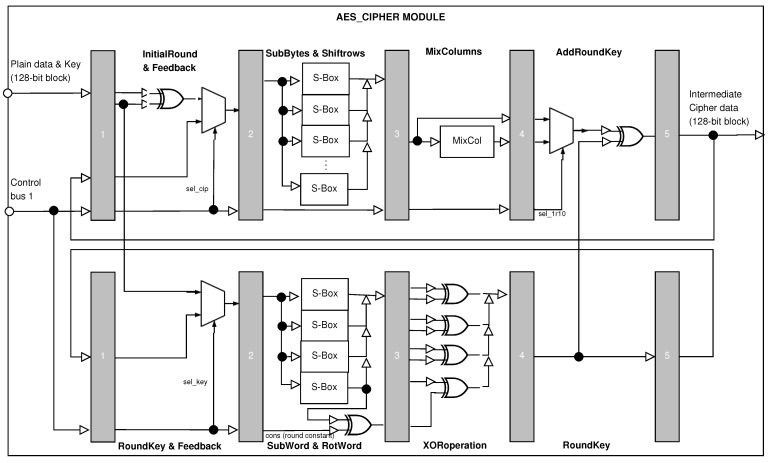
Block diagram of the AES Cipher Module.

The AES_Cipher module presents a pipelined structure divided into five stages, and each one is designed to balance the critical path. This module computes the two main processes defined by the FIPS 197 standard [22]: (1) ciphering process (see the top view in Figure 2) and (2) key expansion (see the bottom view in Figure 2).

Next, the stages of the *AES_Cipher* module will be detailed, describing their specialized operations for both ciphering process and key expansion.

- First stage:
  – Ciphering process: *InitialRound and Feedback*. In this stage, the state is selected from (a) initial round or (b) feedback state. The first stage performs two activities, either computing round 0 or using feedback from all ten rounds. Round 0 is the XOR binary operator between the plain data block and the key block, while the following rounds are the four operations defined by the standard.
  – Key expansion: *RoundKey and Feedback*. This is selected the key in an external way or from the feedback process, where the latter generates round keys.
- Second stage:
  – Ciphering process: *SubBytes and ShiftRows*. In this stage, the state is modified by two transformations: SubBytes and ShiftRows. The first is computed by using S-Boxes, while the second is executed by rearranging the data buses.
  – Key expansion: *SubWord and RotWord*. In this case, the SubWord transformation uses S-Boxes, and the RotWord performs a cyclic permutation. Additionally, an XOR operation is computed using a constant round.
- Third stage:
  – Ciphering process: *MixColumns*. In this stage, the state is modified by the MixColumns transformation, operating on the column-by-column, treating each column as a four-term polynomial, and making multiplications. There are two main outputs of this stage. One is when the MixCol sub-module computes MixColumns, and the other is the direct input from the previous stage. These are necessary because rounds from 1 to 9 use that sub-module, and round 10 does not use it.
  – Key expansion: *XORoperation*. At this point, several XOR operations are executed for providing the round key.
- Fourth stage:
  – Ciphering process: *AddRoundKey*. The multiplexer selects states from the ShiftRows transformation or from the MixColumns transformation through control signal *sel_1r10*. After this, an XOR binary operation is computed using a round key of the key expansion.
  – Key expansion: *RoundKey*. The round key is only stored and sent to the ciphering module.
- Fifth stage:
  – Ciphering process: *Output*. In this stage, the intermediate cipher data are generated, set in the main module’s output, and fed back. The signal *Ready* indicates when the output is correct.
  – Key expansion: *Output*. Unlike the ciphering process in this stage, the round key is not set in some output, but it is fed back.

This module produces five cipher data blocks for the same plain data block, which are sequentially generated and sent to the *Voting* module.

### 4.2. Voting Module

**Figure 3:**
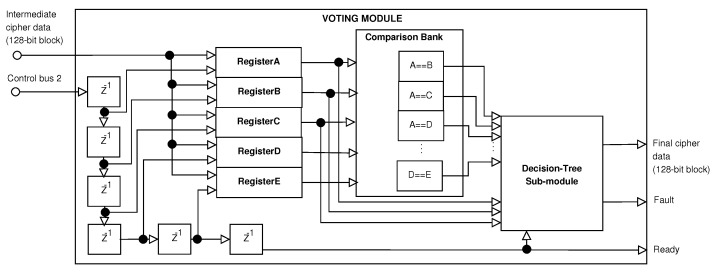
Block diagram of the Voting Module.

The *Voting* Module is shown in Figure 3: (1) it stores *Intermediate Cipher Data* in the five registers, which brings data from the five pipeline stages of the *AES_Cipher* module at different times; (2) it generates comparison signals, where the registers are compared to each other see (*Comparison Bank*); and (3) it uses a *Decision Tree* sub-module for providing the *Final Cipher Data* and the flag Fault for indicating if the *Final Cipher Data* is correct or not.

The decision to determine if there are correct cipher data for the plain data block depends on how many registers, (*RegisterA* to *RegisterE*), are equal, where the majority wins. Firstly, we implemented ten comparators to evaluate if two registers have the same intermediate cipher data (see Table 1).

The comparator’s outputs must be evaluated in a straightforward way. Therefore, there are $2^{10}$ combinations, and the problem of knowing if there are more registers with the same data becomes complex. If we implement this logic, then a bigger memory with 1024 locations of 1 bit will be required.

Secondly, the flag Fault and output *Final Cipher Data* are generated according to the comparisons of the intermediate cipher data from each stage, which are stored in the five registers (see Table 2). This last shows that comparator A==B evaluates RegisterA and RegisterB (intermediate cipher data for the first and second stage, respectively), and it is high if both registers are equal and low if they are different. Other examples are the comparators C==E and D==E evaluating RegisterC versus RegisterE and RegisterD versus RegisterE, respectively.

In this way, Table 2 presents the truth table for generating signals *Fault* and *Final Cipher Data*. The first is high when three or more intermediate cipher data blocks are different (evaluating majority) or when some inconsistency is obtained (see case 1023, where A==D and A==E, but D==E, which is not possible). The second selects some data of the five registers, from RegisterA to RegisterE, which will be the FinalCipherData output bus from the proposed hybrid architecture.

For example, in combination 948, A==B, A==C, A==D, B==C, B==D, and C==D are high and A==E, B==E, C==E, and D==E are low, indicating that RegisterA to RegisterD are equal, but RegisterE is different, and generating that Fault is low (there is an error, but the architecture has recovered from that fault), and the FinalCipherData can be some register from RegisterA to RegisterD. In another example, in combination 2, all comparators are low except for the last one, which is high, indicating that all registers are different, but RegisterD and RegisterE are equal, and generating that Fault is high (there are several errors, and the architecture could not recover from the errors), and the FinalCipherData can be some register, but it is not correct, and the Fault indicates this. In another example, in combination 455, A==C, A==D, A==E, C==D, C==E, and D==E are high and A==B, B==C, B==D, and B==E are low, indicating that RegisterA and RegisterC to RegisterE are equal, but RegisterB is different, and generating that Fault is low (there is an error, but the architecture has recovered from that fault), and the FinalCipherData can be some register from RegisterC to RegisterE or RegisterA. Finally, in case 1024, all registers have the same data, Fault is low, and FinalCipherData can be some register.

A certain level of inner fault-tolerance is provided by the proposed architecture, as is shown in previous examples. This means that if from five blocks of *Intermediate Cipher Data*, one or two are different, then a fault has occurred, and the architecture can detect this error and recover from it. Therefore, the Fault output signal is low, and the *Final Cipher Data* have valid cipher data. Suppose three or more sets of *Intermediate Cipher Data* are different. In this case, the architecture cannot recover from it, the Fault output signal is high, and the FinalCipherData are the data of some register and must be ignored. Moreover, some situations cannot be reached, and they are indicated as inconsistencies. Therefore, the signal Fault is high, and the output FinalCipherData do not matter and can be some register. Finally, if all *Intermediate Cipher Data* are equal, then no error is found, the Fault output signal is low, and the FinalCipherData are correct and can be some register.

The complexity of the logic is very high, and a significant amount of memory is required. In this article, we propose a module based on a decision tree to reduce the complexity and resources.

#### 4.2.1. Predictive Modeling: Decision Trees

In order to reduce the complexity, instead of using all combinations, we propose a decision tree enabling the selection of one output if this is possible according to the comparator’s signals. Shmueli [46] defines *predictive modeling* as “the process of applying a statistical model or data mining algorithm to data for the purpose to predict new or future observations”. Some predictive models include the clustering model, random forest, K-Means, and decision trees.

A decision tree (DT) is a classifier expressed as a recursive partition of the instance space; it classifies instances by sorting them from the root to some leaf node [47]. Typically DTs are implemented in software, but some DT hardware implementations also have been proposed; for example, Fularz et al. in [48] implement a hardware architecture to classify uniform local binary patterns (ULBP). Hardware architectures for performing ensembles of axis-parallel, oblique, and nonlinear decision trees (DTs) are presented by [49].

#### 4.2.2. Decision Tree Sub-Module

**Figure 4:**
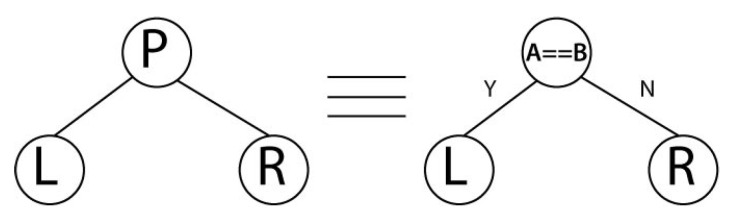
The decision tree *P* for evaluating comparators.

**Figure 5:**
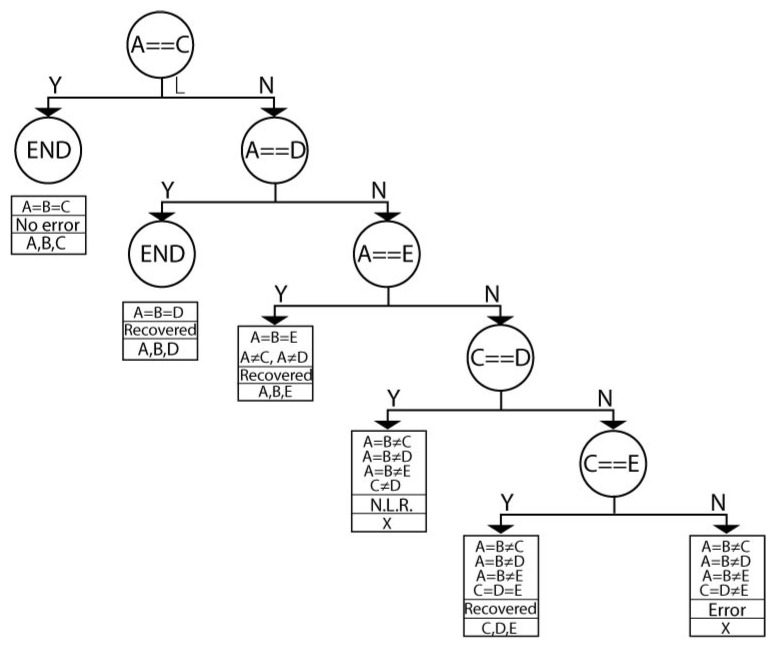
Sub-tree *L* for evaluating the remaining comparators according branch *Y* from tree *P*.

**Figure 6:**
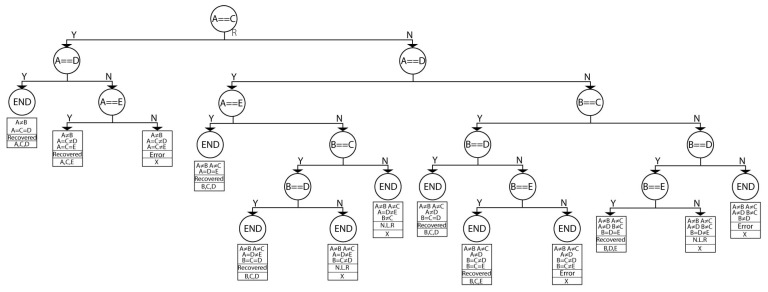
Sub-tree *R* for evaluating the remaining comparators according to branch *Y* from tree *P*.

Figure 4 shows the tree *P* used in this work and is conformed by two sub-trees named *L* and *R* (Figure 5 and Figure 6, respectively). This tree aims to decide and determine that at least three out of five comparisons are necessary. The order of the data arrival is unknown, as well as the arrival possibility. For example, if three comparisons are equal, the the process is finalized. Still, if two comparisons are equal and one is different, then another comparison needs to be done, and if it is equal, it is not necessary to evaluate any more. If the remaining comparisons are different, then a correct output is not reachable, and an error signal is generated.

The first node of the tree *P* considers the comparator A==B. If the *A* and *B* comparators are equal, then branch *Y* (branch *Y*) is chosen, and the sub-tree *L* is selected; if not, branch *N* (branch *N*) is chosen, and sub-tree *R* is selected.

The first node of the *L* tree evaluates A==C. If this happens (branch *Y*), then *A* is equal to *B* and is equal to *C*, and the tree has ended (node END), there is no error, and the encrypted data can be any *A*, *B*, or *C* record. If this does not happen (branch *N*), then it is necessary to continue examining. The next node evaluates A==D. If this is correct, then A=B=D neqC, an error occurs, and the architecture can recover. If not, further evaluations are required. It is important to clarify that other comparisons are not executed because they can be reached; for example, if A==B and A neqC, then the B==C operation is not necessary because it can no longer occur.

In the second sub-tree case, *R* has a complex structure because we must execute more comparisons. *R* mainly considers when A≠C, new branches appearing, always evaluating at least three registers with the same content.

### 4.3. Control Module

**Figure 7:**
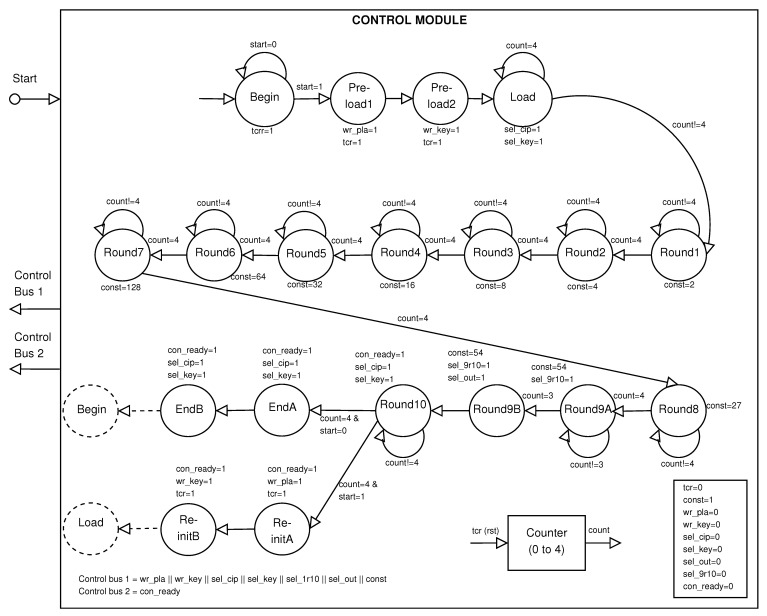
Block diagram of the Control Module.

The *Control Module* carries out the data flow, enabling each process in the *Processing Module* (see Figure 7). This module is based on deterministic finite automatons.

The logic of the ControlModule is the pre-loading and loading of the block inputs (128-bit data block and 128-bit key block), filling the pipeline for input registers. This occurs at the states from Pre−loadA to LoadC. Afterward, the ten rounds are computed (states Round1, …, Round10). In these states, we generate different constants. The AES algorithm [22] requires that the first nine rounds execute the four main operations, whereas the tenth round executes only three operations, and this last is controlled by the Round9A and Round9B states, which avoid the fourth transformation in the tenth round through signal sel_9r10. Finally, the state Round10 evaluates if there are remaining plain data blocks, selecting to go to the state EndA when ciphering has finished or to the state Re−initA when ciphering continues (see Figure 7).

The ControlModule manages: (a) the round constant for the key expansion, (b) the multiplexer for selecting initial or intermediate cipher data blocks (state for rounds from 1 to 10) using sel_cip, (c) the multiplexer for selecting initial or round keys using sel_key, (d) the registers and multiplexer for storing the five final cipher data blocks in the elements from RegisterA to RegisterE and $Z^{-1}$, (e) the counter for the transformations defined by the standard in each state, and f) the multiplexer for avoiding the third transformation in the tenth round through signal sel_9r10.

## 5. Implementation Results and Comparisons

This section describes two different results: (1) evaluation of test vectors for the hybrid architecture with fault detection and (2) analysis of the implementation results on the FPGA and comparisons against related works.

### 5.1. Hardware Architecture Analysis

The analysis and design of hardware architecture are extensive. It is necessary to focus and specify a set of objective metrics for optimization issues. These metrics are selected according to the requirements to be satisfied that require solving a given problem. In this case, this focuses on a cryptographic implementation that takes advantage of a hybrid pipelined structure, which must be measured and compared with iterative and parallel structures.

An integrated circuit or block (*B*) that consumes a power *P*_1_ (*t*), which leads to a need of energy *W*_1_ (*t*), can be calculated as follows:

(1) $$\begin{array}{c} P_{1}\left(t\right)=p\\ W_{1}\left(t\right)=\int P_{1}\left(t\right)dt=\int pdt=pt+c_{1} \end{array}$$

**Figure 8:**
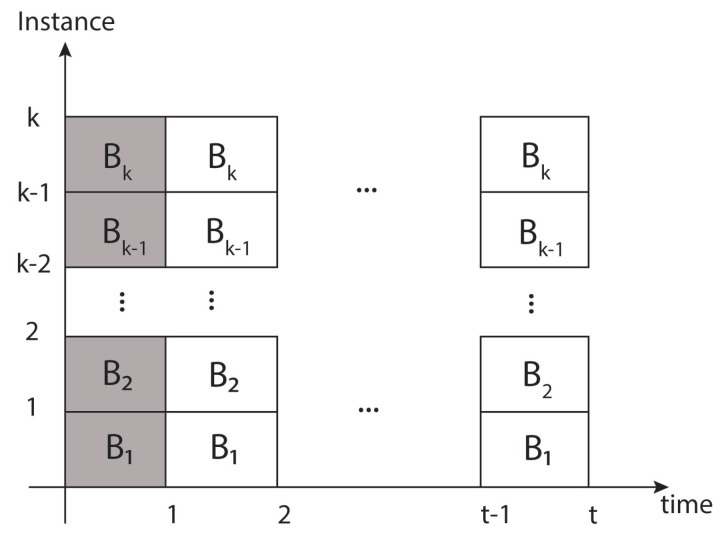
$B_{i}$ blocks are processed in parallel form during one given latency.

The power is constant for an instant and over time, while the energy consumption is variable (*pt* + *c*_1_) according to time. From these measurements, three analyses are performed:

**Architecture for hardware redundancy.** In this architecture, $B_{i}$ blocks are used or replicated to implement the hardware redundancy scheme (blocks $B_{1},\ldots ,B_{k}$) for 1<i<k, which leads to parallel data processing, as shown in Figure 8.

In this scheme, energy and power consumption are sacrificed since all $B_{k}$ blocks are present, so the power and energy consumption is proportional to *k*, as observed below:

(2) $$\begin{array}{c} P_{hw}\left(t\right)=kp\\ W_{hw}\left(t\right)=\int P_{hw}\left(t\right)dt=\int kpdt=kpt+c_{hw} \end{array}$$

**Figure 9:**
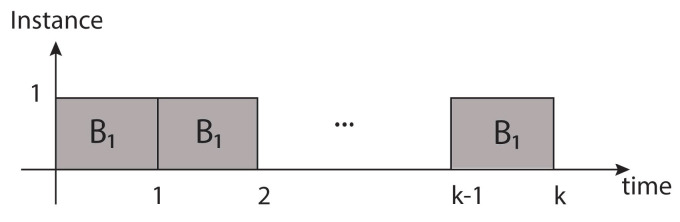
One block ($B_{1}$) is processed in sequential form during *k* latency’s time.

**Temporary redundancy architecture.** In this architecture, a single block *B* will be used *k* times to process a single block of data within a temporary redundancy *k*, reflected in the block ($B_{1}$) from time 1 to time *k* (each of these time segments represents the latency to process a single block of data). See Figure 9.

In this way, a single instance leads to consuming power *p*, where the consumed energy will proportionally increase as time passes, represented in Equation (3). The advantage here is that there are no *k* instances to affect proportionally as in the previous case.

(3) $$\begin{array}{c} P_{ti}\left(t\right)=p\\ W_{ti}\left(t\right)=\int P_{ti}\left(t\right)dt=\int pdt=pt+c_{ti} \end{array}$$

**Figure 10:**
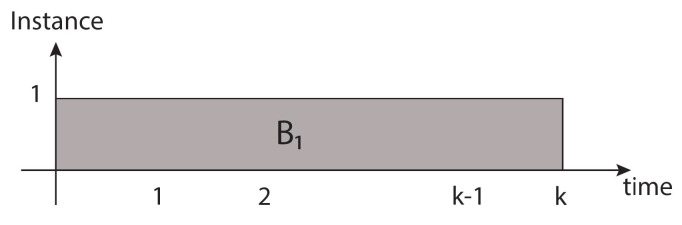
One block ($B_{1}$) is used during a latency, which requires *k* clock cycles.

**Hybrid redundancy architecture.** Finally, for the proposed hybrid architecture, the same instance is used to process *k* blocks of data as in the two previous cases. Unlike the first case, there is only one instance, and unlike the second case, the same block ($B_{1}$) is not occupied *k* times, but only once, with a latency proportional to *k*, as shown in Figure 10.

Likewise, it can be considered that there is a power consumption *p*, where the energy consumption is reflected by the time it takes to process a data block.

(4) $$\begin{array}{c} P_{hy}\left(t\right)=p\\ W_{hy}\left(t\right)=\int P_{hy}\left(t\right)dt=\int pdt=pt+c_{hy} \end{array}$$

The consumption of this scheme seems to be similar to case 2. Still, there are several advantages: data do not travel in long paths, since the internal processes are shorter (which is reflected in the critical path or the minimum clock period), that is, the proposed architecture consumes energy similar to case 2, with the advantage that it will have smaller critical paths. Regarding case 1, there is a lower energy consumption at a lower performance cost, but the passive and active energy is maintained, even if the instances or blocks had a standby process. However, if they had to be put in a standby state, there would not be much need to have several instances of the block so that the architecture would have better results for the vast majority of applications.

In this sense, performance measurement is essential, calculated by the amount of data (a single data block called data): for example, the number of bits in a data block multiplied by the amount of time it takes to the architecture to process it. This is the latency (the number of clock cycles to process a single block of data) multiplied by the time it takes for each clock cycle (period according to the clock frequency). In general, for block *B* issues, it has to be the same data block size (*data*) and the same latency (*latency*), so this relationship can be considered as a constant value (*cte*), and each implementation of the architecture leads to having a value other than the critical path defined by the clock period. This is reflected in the following Equation (5):

(5) $$throughput=\frac{data}{latency*period}=\left(\frac{data}{latency}\right)\frac{1}{period}=\left(cte\right)\frac{1}{period}$$

In this sense, in case 1, a block of data can be processed at a time (within its latency) using all *k* instances, so the performance is the highest of the three cases. In case 2, it has a lower performance than the proposed architecture because it has a shorter critical path (shorter period, better performance).

**Figure 11:**
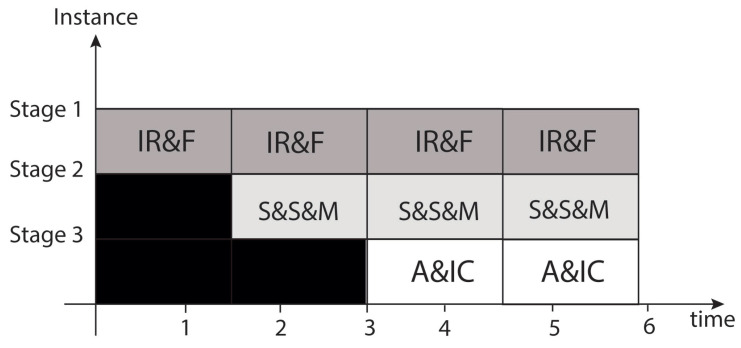
Temporal diagram of the 3-stage pipeline for the AES implementation.

We evaluated two pipeline structures that include the voting system for correcting and detecting errors focusing on an even number of stages. On the one hand, the three-stage design considered integrating different operations within each stage, which can be summarized as follows (see Figure 11):

Stage 1 is IR&F, which is InitialRound and Feedback;

Stage 2 is S&S&M, which is SubBytes, ShiftRows, and MixColumns;

Stage 3 is A&IC, which is AddRoundKey and Intermediate Cipher Data.

**Figure 12:**
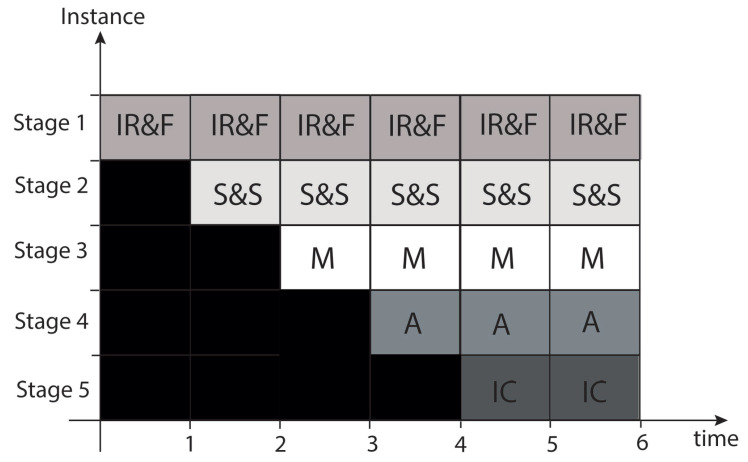
Temporal diagram of the 5-stage pipeline for the AES implementation.

On the other hand, the five-stage architecture design is made up of the following stages (see the Figure 12):

Stage 1 is IR&F, which is InitialRound and Feedback;

Stage 2 is S&S, which is SubBytes and ShiftRows;

Stage 3 is M, which is MixColumns;

Stage 4 is A, which is AddRoundKey;

Stage 5 is IC, which is Intermediate Cipher Data.

We implemented both structures, but a comparison of their implementation results is not shown in this paper due to the large amount of data needed to be added (tables and figures). Nevertheless, we show that the stages do not reduce the paths of each stage; likewise, it is also noted that the path is unbalanced for the operations of each stage of the structure with three stages, where the first stage requires less time than the other two stages. Still, since we must establish a common clock, we cannot improve the path of the others. In this sense, the five-stage structure better balances operations, reducing internal operations and balancing the five-stage path.

According to the analysis, the five-stage structure considers that the latter are filled with data according to the established segmentation. We observe that before starting, all the buffers are empty, and the first time, it is filled with the first block 1; for the second time, the output of the first stage is the input of the second stage, and the first stage is filled with the second input block (repeated input data block). This input block will be entered five times in the architecture during the first five clock cycles; afterward, the blocks are processed iteratively according to the specifications of the AES algorithm [22].

The five-stage hybrid architecture generates five outputs to be compared for detecting and correcting the errors, leading to the five results being saved in a log. To decide if there is an error and evaluate if we can correct it, we must compare those five records. We compare the first record with the remaining four, then the second record with the remaining three, followed by the third record compared with the outlasting two. Finally, the fourth record is compared with the last record. Hence, the number of comparisons is

(6) comparisons=4+3+2+1=10

Then, we need to determine the output through a truth table that involves the ten comparisons; if the output is incorrect, all comparisons were different, or if it is correct, the majority wins: either all the records are equal or at least three records are equal. Thus, the truth table has

(7) $$LogicalCombinations=2^{comparisons}=2^{10}=1024$$

Therefore, the implementation of the truth table requires, for example, a memory of 10 inputs and 1 output, where the spatial computational complexity rises by considering only five records. To solve this computational problem, we evaluated the design of a decision tree to reduce complexity. The tree’s construction is reduced by applying the paradigm “majority wins”; this is enough to decide if the encryption output is correct or incorrect with three equal records. Hence, the number of comparisons is reduced to

(8) $$LogicalCombinations=2^{comparisons}=2^{7}=128$$

### 5.2. Analysis and Evaluation of the Proposed Architecture

In this section, the hybrid pipeline hardware architecture is analyzed and evaluated by using different metrics. It is essential to highlight that the analysis is focused on making fair comparisons between AES architectures with and without capacities of detection and correction of errors.

The hardware architecture is designed using VHDL and is placed and routed on four FPGA technologies: Spartan-7, Artix-7, Kintex-7, and Virtex-7. The tool used is Vivado 2019.2. It is necessary to evaluate how the architecture behaves within different FPGA technologies since it provides information on better technology. According to the FPGA design literature, each technology is quite different, as mentioned in [50], where logic blocks can have different structures regardless of whether they are from the same family or another family, even though the technologies are from the same manufacturer. Commercial processors, such as [51], may have some built-in fault tolerance scheme focused on some logical blocks, which is complex to analyze since the calculation is carried out within a Von Neumann architecture. Hence, the execution of a code is through a general-purpose processor. Next, we present some metrics to evaluate the FPGA performance.

#### Metrics Evaluation Analysis

On the one hand, we provide the *area* utilized by the hardware in terms of look-up tables (LUTs) or a configurable logic block (CLB) slice. The most popular are: *Number of Four-Input LUTs, Number of Slices, Number of Slice Flip Flops, Number of Slice Registers, and Number of IOBs* [52].

On the other hand, we use the next metrics when the specific architecture is implemented on a given FPGA technology. *Frequency* is the clock frequency of the implementation on the FPGA, while *Power Consumption* is the necessary energy for computing the different required processes. The two previous metrics are provided by each tool for implementing designs, whereas the next two metrics are computed by using data from

- Tools such as clock frequency, area, or power;
- Designs such as latency or size of the data blocks.

In this way, *Throughput* and *Efficiency* (throughput-to-area ratio) can help compare different implementations on diverse FPGA technologies. The throughput is calculated using Equation (9), whereas the efficiency is calculated with Equation (10).

(9) $$Throughput=\frac{Data\_block\_size}{\left(Clock\_time)(Clock\_cycles\right)}$$

(10) $$Efficiency=\frac{Throughput}{Number\_of\_Slices}$$

These metrics are used to evaluate the proposed hardware architecture and compare it against related works. Next, three types of analyses about results are made: (1) analysis between 128-bit AES simple hardware architecture without fault detection and 128-bit AES hybrid hardware architecture with fault detection, showing the evolution of this work; (2) analysis for comparing against related works; and (3) analysis for evaluating test vectors.

**Figure 13:**
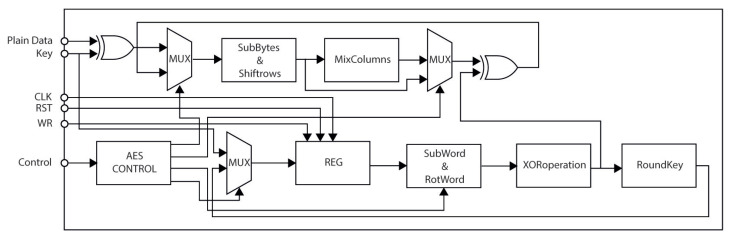
Block diagram of the simple hardware architecture 1R without fault detection or pipeline structure.

For the first analysis, the AES simple hardware architecture has an iterative structure (see Figure 13). It processes a 128-bit plain block during ten clock cycles. This architecture has an unrolled round used iteratively for computing the ten rounds defined in the standard; more details on this architecture are found in [53]. This last architecture is a basic element for designing and developing the AES hybrid-redundancy architecture for fault detection, which has a five-stage pipeline structure.

This first analysis focuses on making fair comparisons between two architectures with and without fault detection and correction, which use comparable designs through similar components. In this comparison, we use the same devices and basic modules, although we also implemented architectures with different goals and structures.

The simple architecture does not detect faults. It processes one 128-bit plain data block at a time because it has an iterative structure, and a final cipher data block for a given plain data block is generated after ten clock cycles.

For the first analysis, Table 3 presents implementation results for the 128-bit AES simple iterative architecture 1R (one round), and Table 4 presents implementation results for the 128-bit AES hybrid-redundancy pipeline architecture with fault detection and correction. These results show: (1) the required amount of hardware resources (numbers of LUT, LUTRAM, FF, IOB, BUFG) and physical requirements (clock period) are provided by the hardware-design tool; (2) characteristics of the design (data size and latency); and (3) characteristics of the implementation (throughput and efficiency), computed by Equations (9) and (10). The reported clock period is the critical path time, which means the minimum clock period, and, consequently, defines the maximum clock frequency (inverse of the minimum clock period).

For all FPGA technologies reported in the Tables, the hybrid architecture 5R (five rounds) occupies approximately 3% to 5% more LUTs, 3.41 times of the number of FFs, and 1 LUTRAM more than the architecture 1R, which are necessary for pipeline registers and additional modules for fault detection and correction.

The hybrid architecture design tries to balance each stage’s paths in the pipeline structure. We add several modules regarding the simple architecture, demonstrated by its critical path time that is shorter. Both architectures process 128-bit plain data blocks, although the latency is different, 11 clock cycles for the simple architecture and 57 clock cycles for the hybrid architecture.

Table 4 presents a throughput that is computed considering a plain data size of 128 bits. However, the data processed by the five-stage pipeline structure during 57 clock cycles are 640 bits (five 128-bit data plain blocks), which is the real size and an excellent advantage of the pipeline architectures. This size is not considered for the proposal of this work. It means that the throughput shown in Table 4 is computed for a data block of 128 bits in the five-stage pipeline structure of the hybrid architecture. The advantage and contribution of this work are that this type of hybrid architecture is used for fault detection and error correction. The throughput of the simple architecture is 4.47, 4.41, 4.613, and 4.83 times more than that for the hybrid architecture for the Spartan-7, Artix-7, Kintex-7, and Virtex-7, respectively.

The throughput of the hybrid architecture is 405, 391, 486, and 479 Mbps, allowing it to be implemented for a significant number of applications. The advantages are focused on similar requirements of hardware resources (LUTs) and clock frequencies, impacting the power consumption. The number of the used FFs increases due to the registers in each stage, where the hybrid architecture requires 3.41 times more FFs than the simple architecture.

The hybrid architecture results show that the design reduces the proportional requirements of hardware resources, and the throughput is slightly less than the proportional value of the simple architecture.

In Table 5, we present the second analysis based on comparisons against related works. Nevertheless, the comparisons are unfair because different FPGA technologies have been used. However, we compare several details about design techniques and implementation results. The related works [39,54] present their hardware architectures on Virtex-5 technology, whereas [40] uses Virtex-II devices.

On the one hand, in [39], a large number of hardware architectures that use the techniques of time and hardware redundancy are reported. The authors use several approaches, from having compact architectures to high performance, but the architectures that interest us directly implement five encryption processes similar to ours. It is interesting to know how the implementation results are detailed since the authors report that their FPGA implementations outperform ASIC implementations for the same architectures, reflected in their performance and frequency of operation, in addition to the area for FRM exceeding the capabilities of the FPGA. Either way, their architectures use 12 clock cycles for FMR (five modular redundancy) and 52 clock cycles for FTR (five-time redundancy), whereas our work requires 57 clock cycles. Although different technologies are used, they report 1886 slices (a slice in Virtex-5 has 4 FFs and 4 LUTs), whereas our architecture uses 1425 LUTs and 1900 FFs, which represents fewer hardware resources. Additionally, the clock frequency is lower than theirs, which is reflected in the reported throughputs. On the other hand, the architectures in [40,54] report schemes based on information redundancy. The first presents several architectures, but, in the table, we only offer two architectures (with or without Power Analysis Countermeasures, PCA). Both lead to having other types of processes, reflected in a large number of modules that require more LUTs and FFs, where the throughput is very low. In the same way, [54] presents an architecture with information redundancy, whose processes are more straightforward than [40], and this is impacted on a greater throughput (2235.22 Mbps) using 430 slices of Virtex-5 technology. The information redundancy focuses on each transformation of the AES implementation. Finally, [41] presents different fault-tolerant architectures implemented on an ASIC and FPGA and uses triple modular redundancy and/or triple time redundancy, generating three values for comparisons. In Table 5 presents implementation results of its best fault-tolerant architecture for AES-128, which requires a 369 MHz clock frequency and few hardware resources, compared with our work, which implements five processes in different architectures, reflected in throughput and hardware resources. However, our work solves a process for comparing $2^{10}$ combinations by using a decision tree.

The implementation of the AES-128 with fault detection and correction uses time, hardware, and information redundancy, which implicates having several AES implementations for hardware redundancy, in order to use the same AES implementations several times or to create additional modules for information redundancy. Our proposal uses a hybrid architecture with fault detection implementing hardware and time redundancies, reached by developing a pipeline structure in the reported hardware architecture. We reduce the number of hardware resources (this amount is close to the hardware resources in a simple architecture—see Table 3 and Table 4) and maintain a large throughput. Future work includes improving this throughput for better efficiency.

### 5.3. Test Vectors for Fault Detection

We evaluate the proposed architecture with 1000 test vectors in a text file, which were applied through the test bench in VHDL at simulation time using the Vivado tool. Each test vector consists of: (1) multiple messages organized on 128-bit blocks, (2) control buses for managing the data flow using control module, (3) the correct cipher data response, and (4) the fault-injection vectors to modify the internal processes and generate internal and external faults.

**Figure 14:**
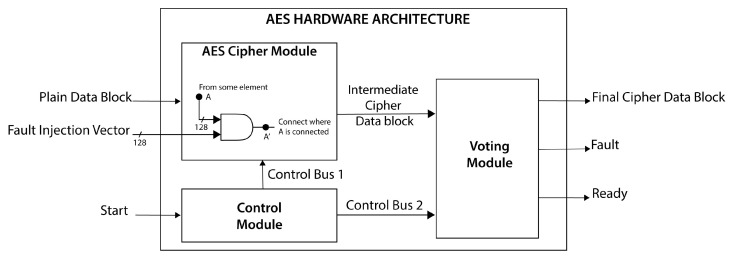
Block diagram of the modified hardware architecture for fault injection. This architecture is only used for testing.

In this evaluation, the key idea includes the fault-injection vector at the previous point 4, used for modifying several computations on diverse hardware modules by applying AND operations and input buses for the hardware architecture. These new buses should not be implemented in the final architecture; this is only for fault-injection testing. The hybrid architecture is modified three times for testing, and they are implemented on the FPGA, but these implementation results are not reported. The three versions are modified by making an AND operation between the 128-bit fault-injection vector and 128-bit intermediate state of the: (a) output of the multiplexer in the first stage, (b) output of the S-Boxes in the second stage, and (c) output of the multiplexer in the third stage (see these modules in Figure 14).

We perform four different runs with these vectors, that is, 4000 tests. The first epoch is one complete presentation of the dataset to be evaluated by the test bench. We applied it to the hybrid architecture without modification, and we did not utilize the fault-injection vectors. The next three epochs are used for the modified hybrid architectures, applying the 128-bit fault-injection vectors for simulating 15,000 faults on the communications. We did the test design through the injection of two error types: stationary and permanent.

In the stationary case, a bit of a data bus is altered in a module through a logical operation (we made a random selection between the logic gates NOT, AND, OR, or XOR) for one, two, three, or four cycles. If it is the NOT operation, nothing else is necessary; if it is the other logical operations in binary form, another input pin of the complete architecture is used to alter the data bus in a single bit. In this way, the injection of errors of the stationary type is carried out with this process. When we apply the NOT operation, the error injection is guaranteed. However, with the other operations, it is not guaranteed (due to the truth tables), with the characteristic that the cryptographic algorithms have the advantage of high diffusion. A small change in a single bit generates a significant difference in a single bit in the output. In the first case, situations are caused where the injection alters one or two registers, and the architecture recovers to 100% since the majority wins. If three or four registers are modified, then the architecture cannot be recovered either, since most registers have different contents, sending the output signals ready but with error, meaning that it did not recover the system. Therefore, it is necessary to recalculate it or not trust the result.

For the permanent case, the injection of permanent errors is generated by using the same logical operations, but with the difference that the alteration is maintained permanently. In this second case, for all the executions, the architecture cannot recover from the error and sends the signals highlighting that a cryptographic output is ready, but the outcome is incorrect, so it can be controlled to return to perform the calculation with this signal. The architecture recovers until any three of the records in the final stage of the pipeline are the same (majority). The failure will persist as long as there is a signal that introduces errors until the architecture generates that majority, and then, in this way, it will be able to recover from the failure.

The results of evaluating stationary and permanent errors in the hybrid architecture enable us to determine 100% of faults.

Finally, we conduct a third analysis based on the injection of temporary and permanent faults (see Table 6). In the first case, the experiments evaluate 5000 test vectors in three epochs (15,000 faults), which modified two stages in two or fewer clock cycles, detecting and correcting 100% of faults and using the modified architecture, where the injection of single errors can affect none, one, or more bits in several modules. The injection did not necessarily modify the output, because the modifications depend on the high cryptographic diffusion and on the bit to be corrupted (if the logical operation affects it or not). In the second case, the experiments evaluate 500 test vectors in three epochs (1500 faults), detecting 100% of faults and correcting 27% of them. The fault injection was applied in three or more clock cycles, which modified, or not, three or more stages registers of the pipeline architecture, depending on the high diffusion, if the bit or bits are modified by the logical operation, or if the content of the three-stage registers or more were affected. This 27% is relative, because if fault injection is applied only in three clock cycles at different pipeline stages, then the correction ratio increases. If this injection increases, then the ratio can decreases. We reached these results by the innovative mechanism proposed, which, at the same time, provides a more reliable fault-detection strategy.

## 6. Conclusions

In real applications, the environment can add undesired signals generated by different sources such as motors, radio frequencies, parasite voltages and currents, movement of the electronic boards, and others. This situation is very critical when we use cryptographic algorithms because they have the avalanche effect, where a slight change in the data being processed provokes the expected output changes significantly. In this way, the characteristics of fault-detection mechanisms are useful for several applications and should have the following features: (a) reliable integrity calculation process, (b) a light computation process, and (c) a fast data validity assessment. Hence, we can apply the proposed hybrid architecture for sectors where an integrity module achieving fault-tolerance is necessary, resulting in new applications such as IoT, IIoT, cloud services, Industry 4.0, and the emerging automotive environment where connectivity is a core part of autonomous and connected cars. This module can be part of greater security schemes or mechanisms to provide other security services such as authentication and digital signatures.

The state-of-the-art redundancy considers managing several replicated hardware systems or using modules implementing information redundancy. In this work, the proposed hybrid architecture is based on time redundancy and hardware redundancy approaches for handling faults, whose five-stage pipeline structure enables the same modules for processing all computations five times, reducing hardware resources and employing the same device. An analysis provided by this article is a fair comparison (see Table 3 and Table 4), where the simple AES architecture is compared with the hybrid AES architecture, using the same FPGA technologies and basic modules. In this analysis, the hybrid architecture requires a similar amount of hardware resources, although its throughput decreases due to the more significant number of clock cycles (latency). We remark on two additional advantages: (1) a similar clock frequency, which does not decrease efficiency, and (2) a similar amount of hardware resources, which does not increment power consumption by not increasing the hardware resources amount. In addition, a new proposal for computing time and hardware redundancies for this algorithm has been made. The throughput satisfies application requirements where throughputs are 0.479 Gbps for Virtex-7 or 0.405 Gbps for Spartan-7.

It is important to highlight that once the encryption calculation has been completed, some systems check errors using CRC (Cyclic Redundancy Check) or hash functions for data transmission or storage. Both functions provide one code only for each message or package. While CRC adds fewer hardware resources and is generally based on XOR operations, hash functions require more hardware resources, since they can use multiplications, tables, memories, registers, logic operations, finite state machines, and several rounds. Our proposal is focused on checking errors while the encryption is being calculated to correct system-level errors or detect faults, avoiding cascade effects and verifying each part of the calculus. In this way, as future work, we will focus on the implementation of lightweight cryptography algorithms used for IoT environments (e.g., SIT, mCrypton, Hummingbird-1, Hummingbird-2, TEA, LED, HIGHT, LBlock, DESL, CLEFIA, PRESENT, TWINE, and RECTANGLE) [55,56], and we will add redundancy schemes for improving hardware requirements. For this implementation, we should consider the generation and integrity of packets for the transport layer of the Open Systems Interconnection model and schemes based on CRCs and hashes.

## Figures and Tables

**Table 1:** Signals of the comparators for each pair of stored cipher data.

Comparator	RegisterA	RegisterB	RegisterC	RegisterD	RegisterE
A==B	X	X			
A==C	X		X		
A==D	X			X	
A==E	X				X
B==C		X	X		
B==D		X		X	
B==E		X			X
C==D			X	X	
C==E			X		X
D==E				X	X

**Table 2:** Truth table for signals.

Combination	A==B	A==C	…	C==E	D==E	Fault	Final Cipher Data
1	0	0	…	0	0	1	None
2	0	0	…	0	1	1	None
…	…	…	…	…	…	…	…
1023	1	1	…	1	0	1	Inconsistency
1024	1	1	…	1	1	0	Some register

**Table 3:** AES hardware architecture 1R.

AES Hardware Architecture 1R				
FPGA technology	Spartan 7	Artix 7	Kintex 7	Virtex 7
Device	xc7s75fgga484-1	xc7a75tfgg484-1	xc7k70tfbg484-1	xc7vh870tflg1932-1
LUT	1382	1381	1353	1355
LUTRAM	0	0	0	0
FF	557	557	557	557
IOB	263	263	263	263
BUFG	1	1	1	1
Clock period (ns)	6.420	6.743	5.190	5.022
Frequency (MHz)	155.7	148.3	192.7	199.1
Data Size	128	128	128	128
Latency (clock cycles)	11	11	11	11
Throughput (Gbps)	1.812	1.726	2.242	2.317
Efficiency (Mbps/LUT)	1.311	1.249	1.657	1.710
Efficiency (Mbps/FF)	3.253	3.098	4.025	4.160

**Table 4:** AES hardware architecture 5R.

AES Hardware Architecture 5R				
FPGA technology	Spartan 7	Artix 7	Kintex 7	Virtex 7
Device	xc7s75fgga484-1	xc7a75tfgg484-1	xc7k70tfbg484-1	xc7vh870tflg1932-1
LUT	1431	1429	1426	1425
LUTRAM	1	1	1	1
FF	1900	1900	1900	1900
IOB	261	261	261	261
BUFG	1	1	1	1
Clock period (ns)	5.542	5.735	4.617	4.679
Frequency (MHz)	180.4	174.3	216.6	213.7
Data Size	128	128	128	128
Latency (clock cycles)	57	57	57	57
Throughput (Gbps)	0.405	0.391	0.486	0.479
Efficiency (Mbps/LUT)	0.283	0.273	0.340	0.336
Efficiency (Mbps/FF)	0.213	0.205	0.255	0.252

**Table 5:** Comparison Table.

Work	Characteristics	Throughput	Approach
		(Mbps)	
[39]—FMR	Xilinx Virtex 5, XC5VLX110T, 1886 slices (409.7%), 244 MHz	2602.60	Fault Tolerant
[39]—FTR	Xilinx Virtex 5, XC5VLX110T, 504 slices (36.2%), 242 MHz	595.60	Fault Tolerant
[40]—w/o PAC	Xilinx Virtex II, XC2V1000, 5191 LUTs, 1628 FFs, 72.21 MHZ	21.40	Fault Detection
[40]—w/ PAC	Xilinx Virtex II, XC2V1000, 5769 LUTs, 1754 FFs, 51.75 MHZ	15.33	Fault Detection
[54]	Xilinx Virtex 5, XC5VFX70, 430 slices, 218.98 MHZ	2235.22	Fault Detection and correction
[41]—AES128	Xilinx Virtex 5, XC5VLX110t, 575 slices, 369 MHZ	1389.17	Fault Tolerant
This work	Xilinx Virtex 7, XC7VH870, 1425 LUTs, 1900 FFs, 213.7 MHZ	479.00	Fault Detection and correction

**Table 6:** Fault injection test results.

Type of Error	Test Vector	Epochs	Detection Ratio	Correction Ratio
Temporary (fixed values applied in ≤2 clk cycles)	5000	3	100%	100%
Permanent (random values applied in ≥3 clk cycles)	500	3	100%	27%

## Abbreviations

The following abbreviations are used in this manuscript:

AES	Advanced Encryption Standard
GPS	Global Positioning System
CRC	Cyclic Redundancy Check
SRAM	Static Random Access Memory
CFTA	Configurable Fault-Tolerant AES
SAFE	Secure AES Frame Encryption
ECU	Electronic Control Unit
V2X	Vehicle-to-Everything
SMS	Short Message Service
SATS	Secure Automotive Telematics Systems
FPGA	Field-Programmable Gate Array
IoT	Internet of Things
IioT	Industrial Internet of Things
IR&F	InitialRound and Feedback
S&S&M	SubBytes, ShiftRows, and MixColumns
S&S	SubBytes and ShiftRows
M	MixColumns
A	AddRoundKey
IC	Intermediate Cipher Data
